# Application value of three-dimensional visualization imaging technology in laparoscopic partial nephrectomy for complex renal tumors

**DOI:** 10.12669/pjms.42.6.14025

**Published:** 2026-06

**Authors:** Gang Li, Yuwei Wang, Weibing Shuang

**Affiliations:** 1 Gang Li, Shanxi Medical University, Taiyuan 030012, Shanxi, China, Department of Urology, Heji Hospital Affiliated to Changzhi Medical College, Changzhi 046011, Shanxi, China; 2 Yuwei Wang, Department of Urology, Heji Hospital Affiliated to Changzhi Medical College, Changzhi 046011, Shanxi, China, Department of Urology, Heji Hospital Affiliated to Changzhi Medical College, Changzhi 046011, Shanxi, China; 3 Weibing Shuang, Department of Urology, First Hospital of Shanxi Medical University, Taiyuan 030012, Shanxi, China

**Keywords:** Laparoscopy, Partial Nephrectomy, Precision Surgery, Renal Tumors, Three-Dimensional Visualization Imaging

## Abstract

**Objective:**

To evaluate three-dimensional visualization (3DV) imaging technology in laparoscopic partial nephrectomy (LPN) for complex renal tumors.

**Methodology:**

In this prospective study conducted at Heji Hospital Affiliated to Changzhi Medical College and First Hospital of Shanxi Medical University between March 2018 and June 2025, 247 patients with complex renal tumors (RENAL score 7-9) were randomized to 3DV preoperative planning group (Group-A, n=123) or conventional 2D imaging group (Group-B, n=124). All underwent retroperitoneal LPN. Group-A received 3D models from enhanced CT for planning and intraoperative navigation. Outcomes included warm ischemia time (WIT), operation time, hemoglobin drop, hospital stay, complications, postoperative rehabilitation, and long-term follow-up.

**Results:**

Group-A vs. Group-B: WIT 20.81±0.94 vs. 27.72±0.82 min (P=0.001); operation time 148.17±5.86 vs. 151.72±5.56 min (P<0.001); hemoglobin drop 10.19±1.00 vs. 16.32±1.41 g/L (P=0.001); hospital stay 6.98±0.15 vs. 11.06±0.86 d (P<0.001). Urinary fistula: 0 vs. 1 case. Analgesic use: 15.6±4.2 vs. 24.3±5.8 mg (P=0.003); ambulation: 22.4±3.6 vs. 34.7±5.1 h (P<0.001); flatus: 28.1±4.0 vs. 41.3±6.2 h (P<0.001). Median follow-up 38 months. Recurrence: 3.3% vs. 4.8% (P=0.54). No difference in creatinine rise (P=0.834).

**Conclusion:**

3DV imaging technology improves LPN precision and safety for complex renal tumors, shortening WIT, reducing bleeding, speeding recovery, and preserving renal function.

## INTRODUCTION

Treatment strategies for T1-stage renal cell carcinoma have fully shifted towards organ preservation, with partial nephrectomy (PN) being collectively recommended by numerous guidelines as the preferred approach for localized tumors ≤ 7 cm.^1^ Compared with radical resection, PN can significantly mitigate the risk of postoperative chronic kidney disease and cardiovascular events without compromising oncology prognosis.^2^ However, for tumors that are completely endophytic or adjacent to the renal sinus or involve multiple segmental arteries/veins, conventional laparoscopic PN must balance the challenges of “precise resection” and “rapid reconstruction”, leading to prolonged warm ischemia time (WIT) and a complication rate (including positive surgical margins and urinary incontinence) of up to 8–15%.^3^-^5^ Given this, preoperative submillimeter-level analysis of the renal vascular, collecting system, and surrounding structures in complex renal tumors is crucial for shortening warm ischemia time and achieving “early unclamping” or “selective ischemia”.

Despite their ability to assess RENAL or PADUA scores, static two-dimensional CT/MRI come with inherent blind spots in describing the anterior-posterior trajectory of the tumor relative to renal vasculature, quantifying the extent of collecting system involvement, and identifying accessory renal arteries.^6^ By contrast, three-dimensional visualization (3DV) technology integrates multi-phase enhanced CT volumetric data and adopts threshold segmentation and surface reconstruction algorithms to simultaneously demonstrate the 4-level branching of the renal artery, venous variations, collecting system trajectories, and tumor topology in a single interactive view, with spatial errors controlled to ≤ 0.8 mm.^7^-^9^ In this regard, retrospective studies suggest that 3DV-assisted laparoscopic PN can shorten WIT and mitigate complications.^10^,^11^ However, available evidence is primarily based on single-center, non-randomized designs, combined with a lack of a unified anatomical definition for “complex renal tumors”, leading to significant heterogeneity in results.

## METHODOLOGY

**Fig.1:**
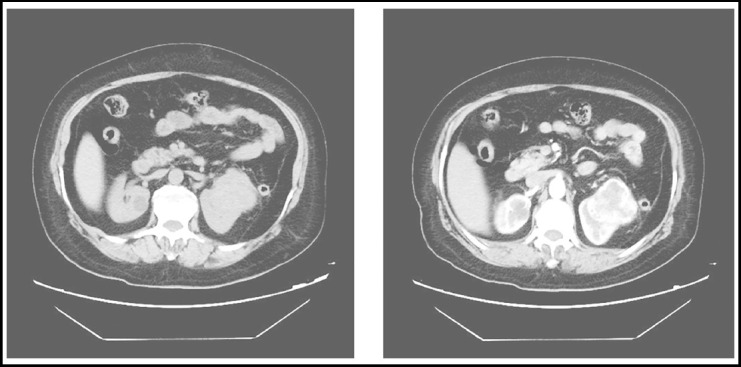
CT Image of Renal Tumor. (A: Irregular density shadow was seen in the left kidney on plain scan; B: There was obvious inhomogeneous enhancement in arterial phase in contrast-enhanced scan.).

In this study, a total of 308 patients with complex renal tumors admitted to Heji Hospital Affiliated to Changzhi Medical College and First Hospital of Shanxi Medical University between March 2018 and June 2025 were prospectively included. Specifically, 308 cases that underwent retroperitoneal laparoscopic partial nephrectomy were identified via a preliminary database screening, with 247 finally included in the statistical analysis (Fig.1) as per a RENAL score of 7–9 points and the definition of clinical stage T1a–T1b N0 M0. Additionally, the subjects were assigned to either the three-dimensional visualization preoperative planning group (Group-A, n = 123) or the conventional two-dimensional imaging control group (Group-B, n = 124) 24 h preoperatively using a computer-generated block randomization scheme (block size = 4).

### Ethical approval:

The study was approved by the Institutional Ethics Committee of First Hospital of Shanxi Medical University (No.:KYLL-2025-068, Date: February 24, 2025), and written informed consent was obtained from all participants.

### Inclusion criteria:

- Patients with single renal parenchymal masses on enhanced CT.
- Patients with clinical stages conforming to T1aN0M0 or T1bN0M0 outlined in the AJCC (8th edition).
- Patients with RENAL scores of 7–9 points.

### Exclusion criteria:

- Patients with multiple renal tumors.
- Patients with preoperative imaging or bone scan suggesting distant metastases.
- Patients with severe cardiac, hepatic, or renal insufficiency (ASA ≥ IV) who were unable to tolerate pneumoperitoneum or renal ischemia.
- Patients with a previous history of ipsilateral renal surgery.

No statistically significant differences were observed in baseline variables such as age, gender distribution, body mass index (BMI), maximum tumor diameter, side of the tumor, RENAL score, preoperative hemoglobin, and serum creatinine levels between the two groups (*P* > 0.05, Table-I), indicating good inter-group comparability.

**Table-I:** Comparison of general information of two groups of patients

Items	Group-A (n=123)	Group-B (n=124)	P value
Age (years)	63.66±1.73	63.79±1.76	0.559
Gender (male/female)	72/51	78/46	0.567
BMI (kg/m^2^)	26.47±0.59	26.45±0.52	0.778
Tumor diameter (cm)	4.21±0.19	4.19±0.17	0.384
Side of the tumor (left/right)	49/74	57/67	0.398
R.E.N.A.L.	7.64±0.20	7.67±0.19	0.228
Preoperative hemoglobin (g/L)	138.91±1.63	138.86±1.59	0.807
Preoperative serum creatinine (μmol/L)	65.38±2.46	65.19±2.53	0.550

***Note:*** There was no significant difference between the two groups, P > 0.05.

### 3D Visualization Model Construction Process:

**Fig.2:**
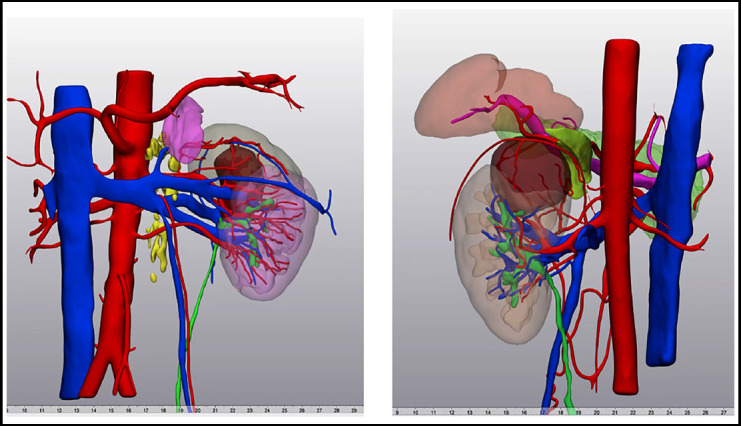
3D Visualization Reconstruction and Intraoperative Navigation Images (A: Completely endophytic tumor B: Large renal tumor. The kidney appears semi-transparent gray, with arteries (red), veins (blue), tumor (brown), and renal collecting system and ureter (green).

All enrolled patients underwent standardized 256-slice CT for renal four-phase scanning (plain scan, cortical phase, medullary phase, excretory phase) with the following parameters: slice thickness 0.625 mm, tube voltage 100 kV, automatic tube current modulation (DoseRight 4D), and DICOM datasets reconstructed at one minute intervals. Afterward, data from Group-A were imported into Mimics Innovation Suite 21.0 (Materialise, Leuven, Belgium) for three-dimensional reconstruction. The specific steps were as follows:

*Image Preprocessing:* Non-local means filter (sigma=0.8) was utilized to reduce quantum noise while preserving vascular edge sharpness;

*Multi-threshold segmentation*: Independent masks were assigned to the renal parenchyma (−30 ~+180 HU), renal arteries (> 220 HU and continuous with the abdominal aorta), renal veins (180~220 HU), collecting system (excretory phase > 300 HU), and tumors (relative enhancement > 20 HU in the cortical phase with clear demarcation from renal parenchyma);

*3D surface rendering*: STL surface models were generated using the Marching Cubes algorithm, followed by Laplacian smoothing (2 iterations, λ = 0.5) to eliminate stair step artifacts;

*Color mapping and transparency adjustment*: The kidney was set to 15% transparency, with tumors (red), arteries (red), veins (dark blue), collecting system and ureter (cyan-green), and Phong shading was enabled to enhance depth perception;

The final model was exported in .m3d format to 3D-PDF and Hololens two mixed reality platform for preoperative multidisciplinary discussions and intraoperative real-time reference. All reconstruction processes were performed by the same imaging technician with five years of modeling experience (Fig.1 and 2). The average time consumed for constructing each 3D model, measured from DICOM data import to completion of the final .m3d format model ready for clinical use, was recorded. The mean construction time was 3.0±0.6 hours. No intraoperative or postoperative events were attributed to delays in model preparation. Reproducibility assessment: To ensure model reliability, we performed the following validation:

- All segmentations were reviewed by an experienced urologist (third author) for anatomical plausibility before clinical use.
- As noted above, intra-observer reproducibility was tested on 20 randomly selected cases.
- No external validation by multiple independent observers was performed, as staffing constraints limited modeling to a single trained technician.

### Laparoscopic Surgical Procedure:

After successful general anesthesia, the patient was placed in the lateral decubitus position on the unaffected side with the waist bridge elevated to 30°. Afterward, routine disinfection and draping were performed, and retroperitoneal access was established with four ports.

A 2- cm transverse incision was made 2-cm below the 12th rib along the posterior axillary line, and blunt dissection with hemostats was performed down to the lumbodorsal fascia, followed by balloon dilation of the retroperitoneal space, and insertion of a 12 mm disposable Trocar as the camera port. 10-mm Trocars were inserted at 2 cm above the iliac crest along the midaxillary line and 2cm below the costal margin along the anterior axillary line as working ports.

A 5-mm auxiliary Trocar was added 2 cm below the midpoint between the anterior and midaxillary lines. Meanwhile, the pneumoperitoneum was set at 14 mmHg, and a 30° laparoscope was directed away from the renal hilum for exposure.

In Group-A (3D navigation group), the preoperatively constructed .m3d-format model was imported into the laparoscopic image processing unit via OpenGL interface, overlaid onto the real-time surgical field with 40% transparency, enabling synchronized visualization of tumor boundaries, three level branching of the renal artery, and the collecting system trajectory. By contrast, Group-B referenced only hanging two-dimensional CT films.

The subsequent steps were consistent in both groups: Gerota’s fascia was incised longitudinally, and the main renal artery was dissected extraperitoneally outside the perirenal adipose capsule, with 8 mm silicone vascular slings placed for identification. In the meantime, the renal artery was occluded using atraumatic bulldog clamps, and timing was initiated. Afterward, a 0.5 cm safety margin was established along the tumor pseudocapsule, with the 3D Group correcting the resection plane in real-time via fused imaging to avoid entering the renal sinus. During basal dissection, the collecting system was identified via the green channel identification, with defects immediately closed with continuous 4-0 Vicryl sutures.

Moreover, the wound was closed in two layers: the medulla was sutured with 2-0 V-Loc barbed sutures in a figure-of-eight pattern for the deep layer, and interrupted suturing of renal parenchyma was performed using 1-0 Vicryl, with needle depth guided by navigation scales to maintain ≥ 4 mm distance from the collecting system. Dynamic Registration and Adjustment Protocol for Organ Displacement: To address intraoperative organ displacement caused by positioning changes, pneumoperitoneum fluctuations, or surgical manipulation, we implemented a multi-layered adjustment strategy. Prior to insufflation, three fixed anatomical landmarks (renal hilum, psoas margin, inferior renal pole) were registered to the 3D model for subsequent reference. Pneumoperitoneum was maintained at 14±2 mmHg, with ventilator adjustments during critical phases to minimize diaphragmatic-induced displacement. When displacement exceeded 5 mm, two-point manual registration (identifying visible structures such as arterial bifurcation and tumor edge) was performed to recalculate overlay alignment, requiring 45-90 seconds and executed 1-3 times per case. The 3D model was displayed at 40% transparency over real-time video to reduce dependency on rigid registration, and auxiliary trocar positioning minimized retraction-induced torque. Warm ischemia time (WIT) was recorded immediately upon clamp release, and fibrin glue was sprayed on the wound surface. A 22 Fr retroperitoneal drain was placed, with the incision closed in layers. The operation time was defined as the duration from skin incision to completion of the final suture.

### Postoperative Rehabilitation Indicators:

The following rehabilitation-related parameters were recorded for both groups:

- postoperative analgesic use, defined as the total dose of parenteral analgesics (morphine equivalents, mg) administered via patient-controlled intravenous analgesia (PCIA) within the first 48 hours post-surgery;
- time to first ambulation, defined as the time (in hours) from the end of surgery to the patient‘s first unassisted walking outside the bed for ≥ 5 meters, as documented by nursing records;
- time to first flatus, defined as the time (in hours) from the end of surgery to the first documented passage of flatus, reflecting gastrointestinal recovery. All rehabilitation indicators were recorded by nursing staff blinded to group allocation.

### Follow-up protocol:

All patients were followed postoperatively at three, six, and 12 months in the first year, then annually thereafter. Contrast-enhanced CT or MRI was performed at six months, 12 months, and annually to assess for tumor recurrence (defined as new enhancing lesion at the resection bed or distant metastasis). Follow-up data were collected through outpatient records and telephone interviews when necessary, with censoring on June 30, 2025.

### Statistical Analysis:

All data were double-validated using SPSS 23.0 (IBM Corp., Armonk, NY) and R 4.2.1. Measurement data were first tested for normality via the Shapiro-Wilk test: if normally distributed with homogeneous variance (Levene’s test, P ≥ 0.10), they were expressed as mean ± standard deviation (*x̄*+*s*), with inter-group comparisons performed using the independent samples t-test; if not normally distributed, they were expressed as median (interquartile range) [M(IQR)] and analyzed using the Mann-Whitney U test. Meanwhile, count data were expressed as number (percentage) [n(%)] and analyzed using the chi-square test or Fisher’s exact test (if expected frequency < 5). Additionally, the effect size of the primary endpoint (WIT) was reported as a mean difference (MD) with 95% CI, and the secondary endpoint (changes in postoperative eGFR) was analyzed using ANCOVA with preoperative eGFR as a covariate. All tests were two-tailed, with the significance level (α) set at 0.05, and multiple comparisons were corrected using the Bonferroni method, with a corrected *P* < 0.05 considered statistically significant.

## RESULTS

Surgeries were successfully completed in both groups, with no conversion to open surgery. In Group-A, the median time for 3D model construction was 3.0 h (IQR: 2.5–3.6 h). Model construction time did not significantly correlate with RENAL score (r = 0.12, P = 0.42) or tumor diameter (r = 0.09, P = 0.58), indicating that tumor complexity did not substantially prolong reconstruction time in this cohort. Statistically significant differences were observed between the two groups in terms of warm ischemia time, decrease in 24-h postoperative hemoglobin, postoperative hospital stay, and operation time (warm ischemia time: *P* = 0.001; decrease in 24-h postoperative hemoglobin: *P* = 0.001; postoperative hospital stay: *P* < 0.001; operation time: *P* < 0.001), with Group-A outperforming Group-B in these parameters. Additionally, there was no statistically significant difference in the increase in 24-h postoperative serum creatinine between the two groups (*P* = 0.834). Notably, one patient in Group-B developed a urinary fistula postoperatively, which was treated with the placement of a ureteral stent on Day three postoperatively and subsequently improved. See Table-II for the comparison of surgical outcomes between the two groups. Postoperative rehabilitation outcomes are summarized in Table-III. Patients in Group-A (3D visualization group) required significantly less parenteral analgesic within the first 48 hours postoperatively compared with Group-B (15.6 ± 4.2 mg vs. 24.3 ± 5.8 mg morphine equivalents, P = 0.003). Moreover, Group-A demonstrated earlier ambulation (22.4 ± 3.6 h vs. 34.7 ± 5.1 h, P < 0.001) and earlier flatus (28.1 ± 4.0 h vs. 41.3 ± 6.2 h, P < 0.001) compared with Group-B. These findings suggest that 3D visualization-assisted LPN facilitates faster postoperative recovery.

**Table-II:** Comparison of surgical outcomes between the two groups.

Items	Group-A (n=123)	Group-B (n=124)	P
Operation time (min)	148.17±5.86	151.72±5.56	<0.001
Warm ischemic time (min)	20.81±0.94	27.72±0.82	0.001
Postoperative hospital stay (d)	6.98±0.15	11.06±0.86	<0.001
Decrease in hemoglobin (g/L)	10.19±1.00	16.32±1.41	0.001
Increase in serum creatinine (μmol/L)	12.57±2.90	12.66±2.00	0.834

**Table-III:** Comparison of postoperative rehabilitation indicators between the two groups

Items	Group-A (n=123)	Group-B (n=124)	P
Postoperative analgesic use (morphine equivalents, mg, 0–48 h)	15.6±4.2	24.3±5.8	0.003
Time to first ambulation (h)	22.4±3.6	34.7±5.1	0.001
Time to first flatus (h)	28.1±4.0	41.3±6.2	<0.001

### Long-term follow-up outcomes:

The median follow-up duration was 38 months (IQR: 18–62 months), with 189 patients (76.5%) completing at least 12 months of follow-up. No patients were lost to follow-up within the first six months. Tumor recurrence occurred in four patients (3.3%) in Group-A and 6 patients (4.8%) in Group-B (P = 0.54). All recurrences were local (resection bed) except one distant metastasis (lung) in Group-B. The 3-year recurrence-free survival rates were 96.5% (95% CI: 92.1–98.6%) in Group-A and 94.2% (95% CI: 89.3–97.0%) in Group-B (log-rank P = 0.38).

Learning curve for 3D navigation. CUSUM analysis of WIT in Group-A showed an initial upward trend during the first 18 cases, followed by a plateau after case 22, and a consistent downward trend thereafter. The inflection point occurred at case 22, indicating that proficiency (consistent WIT below 25 min) was achieved after approximately 22 cases. When comparing the initial 22 cases (learning phase) with the subsequent 101 cases (proficient phase), mean WIT improved significantly from 24.6±1.8 min to 19.9±1.1 min (P < 0.001). No significant difference in complication rates was observed between the two phases (P = 0.64).

## DISCUSSION

Partial nephrectomy is the gold standard for localized renal tumors due to its advantages in reducing CKD and improving survival.^12^ However, for complex tumors (hilar or completely endophytic), LPN faces challenges including prolonged WIT, increased blood loss, and collecting system injury, which compromise surgical safety and renal recovery.^13^ Thus, improving surgical precision for complex LPN is a key research focus. The success of such surgery depends on accurate preoperative anatomical assessment.^14^ Conventional 2D CT/MRI lacks intuitive 3D spatial visualization, making it difficult for surgeons to identify tumor location, blood supply, and proximity to critical structures during LPN.^15^ Misjudgment can cause vascular injury, positive margins, or excessive resection. In this regard, 3D visualization technology reconstructs CT data into a multi-structural model with color-labeled differentiation of tumors, vessels, and collecting systems. The model supports interactive operations such as rotation, scaling, and virtual resection, allowing surgeons to assess tumor volume, hilar distance, vascular variations, and parenchymal defects. Based on these details, individualized surgical plans can be developed to minimize damage and optimize outcomes.^16^,^17^ In this study, Group-A (3D visualization-assisted LPN) reported an incidence of 0% for urinary fistula compared with one case in Group-B, along with a significantly shorter postoperative hospital stay (6.98±0.15 d vs. 11.06±0.86 d, *P* < 0.001), highlighting the clinical value of 3D preoperative planning in mitigating anatomical injuries and accelerating postoperative recovery. Warm ischemia time (WIT) is a critical factor influencing renal function recovery following LPN, while complex tumor anatomy often leads to prolonged renal artery occlusion time, thus increasing the risk of postoperative renal function impairment.^18^ To address this, 3D visualization technology provides an effective solution through intraoperative navigation: the integration of 3D models with laparoscopic systems enables real-time matching of virtual images and actual anatomical structures, which facilitates rapid identification and occlusion of renal arteries (including accessory renal arteries), reducing the risk of incomplete occlusion or positional deviation.^19^ Therefore, Group-A demonstrated a markedly lower decrease in postoperative hemoglobin (10.19±1.00 g/L vs. 16.32±1.41 g/L, *P* = 0.001). Moreover, 3D models guide surgeons to precisely resect tumors along preset margins, minimizing positive surgical margins and unnecessary renal parenchymal loss, while aiding in the identification and repair of collecting system injuries, so as to prevent urinary complications. Our learning curve analysis suggests that approximately 22 cases are needed for a surgeon to achieve proficiency in 3D visualization-assisted LPN, based on WIT as the primary endpoint. This is comparable to the learning curve reported for standard LPN (15–25 cases) . For hospitals planning to adopt this technology, we recommend that the first 20–25 cases be performed by an experienced laparoscopic surgeon with dedicated technical support for model alignment. After this learning period, the WIT and complication rates are expected to stabilize at levels superior to conventional LPN.

Notably, WIT in Group-A was remarkably shorter than in Group-B (20.81±0.94 min vs. 27.72±0.82 min, *P* = 0.001), demonstrating the efficiency of 3D navigation. Particularly, the shorter WIT in Group-A contributes to superior long-term renal function preservation, consistent with the principle of “precision resection and functional protection”. The advantages of Group-A regarding WIT, blood loss, and hospital stay indicate that 3D technology not only shortens operation time but also substantially improves surgical quality. Compared with conventional LPN, 3D visualization technology demonstrates significant advantages in both short-term and long-term outcomes, with the short-term benefits including reduced bleeding, shorter hospital stay, and lower incidence of complications such as urinary fistula. The long-term follow-up data provided in this study, suggests that shortening WIT and preserving renal parenchyma may mitigate the risk of CKD development, which is closely associated with overall survival.^20^

In the research by Kälble S et al.^21^ on the relationship between acute kidney injury (AKI) and chronic kidney disease (CKD) progression following partial nephrectomy, 631 patients with T1–T2 renal tumors who underwent robotic or laparoscopic partial nephrectomy from 2010 to 2022 were retrospectively enrolled, in which AKI was defined as per the RIFLE criteria, with a median follow-up of 5.3 years. Their findings suggested a postoperative AKI incidence of 23.6%, among which 42.7% developed CKD progression within one year (eGFR decline ≥ 25% or stage escalation). Multivariate Cox analysis confirmed AKI as an independent risk factor for CKD progression (HR = 2.38, 95%CI: 1.74–3.26), significantly associated with cold ischemia time, renal parenchymal resection volume, preoperative eGFR, and gender.

In this study, a nomogram incorporating these variables was further constructed, in which an internal validation C-index of 0.81 may indicate its ability to accurately predict individual risk of postoperative CKD progression, thus providing an evidence-based basis for screening high-risk populations and optimizing perioperative renal protection strategies.^21^ Surgical outcomes of conventional LPN are highly dependent on the experience of surgeons, resulting in significant variability in prognosis. By contrast, 3D technology enables reproducible preoperative planning and intraoperative guidance, helping narrow the technical gap among surgeons and facilitating the popularization of complex LPN techniques to less experienced medical centers.

The superior postoperative rehabilitation outcomes observed in Group-A namely, reduced analgesic requirement, earlier ambulation, and earlier flatus—can be attributed to several factors enabled by 3D visualization technology. First, the shorter warm ischemia time (20.8 ± 0.9 min vs. 27.7 ± 0.8 minutes) and reduced intraoperative blood loss (reflected by smaller hemoglobin decrease) likely minimized renal and systemic inflammatory responses, thereby alleviating postoperative pain and facilitating early mobilization. Second, the precise preoperative planning allowed for more accurate resection plane determination and collecting system repair, which may have reduced postoperative peritoneal irritation and ileus. Notably, all 3D models in Group-A were constructed by a single imaging technician with consistent expertise, ensuring model quality without inter-operator variability. This standardization, combined with the intraoperative navigation derived from these models, enabled surgeons to perform more predictable and less traumatic dissections, ultimately translating into faster recovery. These findings align with previous reports that 3D technology improves not only surgical precision but also patient-reported recovery outcomes.

### Limitations:

Despite the broad application prospects of 3D visualization technology, attention shall still be paid to several limitations. First, the accuracy of models depends on CT resolution and may be affected by intraoperative respiratory motion or organ displacement, making manual registration necessary, which is a cumbersome process that cannot achieve complete precision. In light of this, the development of an automated dynamic fusion system with real-time adjustment is a key direction for future research. Second, the need for specialized software and professional technicians leads to increased costs and operational complexity, thus limiting its application in resource-limited regions. Given this, simplifying workflows and reducing costs are deemed essential for the widespread promotion of this technology. Third, the current applications of this technology are primarily centered on surgical planning and navigation, while future integration with artificial intelligence (AI) is expected to enable functions such as automatic segmentation, margin prediction, and postoperative assessment. Meanwhile, the combination of 3D models with multimodal imaging techniques, such as magnetic resonance imaging (MRI) and contrast-enhanced ultrasound, may further enhance the clarity of anatomical structure visualization. Regarding clinical feasibility, the observed model construction time (median 3.0 h) is acceptable for elective complex renal tumor surgeries when performed one day prior to operation, as it does not prolong the inpatient waiting period beyond routine preoperative preparation. However, to further enhance scalability—particularly in high-volume centers or emergency settings—future efforts should focus on reducing this time cost through automated segmentation algorithms. Additionally, most research on 3D-guided LPN, including this one, is conducted via single-center studies with limited sample sizes. Therefore, multicenter, randomized, and controlled trials with long-term follow-ups are required in the future, so as to validate the effects of this technology in renal function preservation, oncological outcomes, cost-effectiveness, and reproducibility.

## CONCLUSIONS

3D visualization technology significantly improves the precision and safety of LPN for complex renal tumors, which shortens WIT, reduces intraoperative bleeding, mitigates complication rates, and preserves renal function by optimizing preoperative planning and enabling intraoperative real-time navigation. Despite challenges in registration accuracy, cost control, and standardization, 3D visualization technology is expected to witness expanded applications in minimally invasive urology thanks to continuous technical improvements and rigorous clinical evaluation, ultimately providing higher-quality medical services to patients.

### Authors’ Contributions:

**GL:** Literature search, conceived and designed the study.

**YW:** collected the data and performed the analysis.

**WS:** Writing of the manuscript and is responsible for the integrity of the study.

All authors have read and approved the final manuscript.
